# “That same stigma...that same hatred and negativity:” a qualitative study to understand stigma and medical mistrust experienced by people living with HIV diagnosed with COVID-19

**DOI:** 10.1186/s12879-021-06693-5

**Published:** 2021-10-14

**Authors:** Andre Hall, Olivier Joseph, Samantha Devlin, Jared Kerman, Jessica Schmitt, Jessica P. Ridgway, Moira C. McNulty

**Affiliations:** 1 University of Chicago, 5801 S. Ellis Avenue, Chicago, IL 60637 USA; 2 University of Chicago Pritzker School of Medicine, 924 E. 57th Street #104, Chicago, IL 60637 USA; 3 University of Chicago Section of Infectious Diseases and Global Health, 5841 S. Maryland Avenue, MC 5065, Chicago, IL 60637 USA; 4 University of Chicago, Chicago Center for HIV Elimination, 5841 S. Maryland Avenue, MC 5065, Chicago, IL 60637 USA

**Keywords:** PLWH, Stigma, Medical mistrust, COVID-19

## Abstract

**Background:**

The COVID-19 and HIV epidemics have exacerbated existing inequities among vulnerable groups and severely impacted communities of color. People living with HIV (PLWH), who may already face stigma or discrimination, are at risk of experiencing further stigma as a result of COVID-19, which can result in medical mistrust.

**Methods:**

We performed qualitative interviews between June and August 2020 among 32 PLWH, including 10 individuals diagnosed with COVID-19. A majority of participants perceived themselves as having an increased risk of contracting COVID-19 due to their HIV status.

**Results:**

Of those who tested positive for COVID-19, the majority regarded their HIV diagnosis as having a more profound impact on their lives but found similarities between COVID-19 stigma and HIV-related stigma. Many participants also expressed mistrust.

**Conclusions:**

These results can be used to better understand the perspectives of PLWH during the COVID-19 pandemic and have important implications for potential COVID-19 vaccine hesitancy and future health crises.

**Supplementary Information:**

The online version contains supplementary material available at 10.1186/s12879-021-06693-5.

## Background

Both the COVID-19 and HIV epidemics have disproportionately impacted vulnerable communities, exacerbating inequities. The COVID-19 pandemic has disrupted lives and medical care for many, particularly people living with chronic conditions such as HIV, and has most severely impacted communities of color. COVID-19 hospitalization and death rates are 2.9 and 1.9 times higher among Black people and 3.1 and 2.3 times higher among Latinx persons, respectively, compared to those of non-Hispanic white people [1]. Moreover, in many U.S. states, racial and ethnic minorities make up a disproportionately high percentage of COVID-19 cases [2]. Similarly, the HIV epidemic in the United States has most impacted communities of color, as Black people represented 13% of the U.S. population but 41% of people living with HIV (PLWH), and Latinx individuals represented 18% of the U.S. population but 23% of PLWH in 2018 [3].

Research has demonstrated that both COVID-19 and HIV disproportionately impact communities of color due to the interplay of several factors related to social determinants of health, including structural racism [4]. A study of 80 U.S. cities found that poverty, unemployment, and vacant housing were associated with a greater HIV incidence in Black communities [5]. Having a low income has been associated with greater exposure to both HIV and COVID-19 [4], whereas people with higher incomes and private insurance have increased access to HIV and COVID-19 testing and treatment [6]. Additionally, Black and Latinx Americans make up a significant percentage of essential workers in the United States [7], with a study estimating that 65% of Latinx households and 57% of Black households had at least one person who could not work remotely during lockdowns associated with the COVID-19 pandemic as compared to 47% of white households [8]. Moreover, intersectional stigma, medical mistrust, and decreased likelihood of referral for HIV or COVID-19 testing results in reduced engagement in the healthcare system among people of color and PLWH [6].

PLWH may face numerous challenges including stigma that can lead to negative social and health consequences. Racial, sexual, and gender minorities are disproportionately impacted by HIV: of new HIV diagnoses in the United States, 69% are given to men who have sex with men (MSM) and 45.4% to Black people [9]. Additionally, the HIV prevalence rate among transgender women is estimated to be 27.7%, even higher than the 25% prevalence rate estimated for MSM [10]. These groups are at a higher chance for experiencing adverse health outcomes due to the intersectionality of these marginalized populations [11, 12]. In addition to poorer health experiences, PLWH may face stigma associated with their HIV status including but certainly not limited to social rejection, verbal insults, and discrimination in healthcare [13]. Perceived community stigma has been significantly associated with internalized stigma, leading to poorer self-esteem, and negatively associated with medication adherence among PLWH [14]. Fear of stigma and discrimination can also inhibit linkage to care and support services needed by PLWH [15].

People diagnosed with COVID-19 have also faced some degree of stigma and discrimination in the U.S. People diagnosed with COVID-19 must isolate for a period of time, facing economic and social disadvantages, and fear of transmission to others. Low-wage workers may miss out on necessary wages or even lose their jobs. In certain communities, fear of receiving a COVID-19 diagnosis and the associated implications has caused some people to avoid obtaining a COVID-19 test despite having symptoms consistent with COVID-19 [16].

Although studies have examined the interaction between COVID-19 and HIV on a microbiological level, few studies have focused on the social determinants of health impacting PLWH’s experiences during the COVID-19 pandemic, from dealing with the uncertainty of their vulnerability to COVID-19 to their feelings of anxiety during the testing process or stigma associated with being diagnosed with COVID-19. This study aimed to examine the perspectives of PLWH regarding potential perceived vulnerability in contracting COVID-19, fears and concerns regarding testing for COVID-19, and comparisons between receiving a COVID-19 diagnosis and HIV diagnosis.

## Methods

We conducted a qualitative study, performing semi-structured remote interviews with PLWH in Chicago from June 2020 through August 2020. PLWH who received medical care at The University of Chicago were recruited to participate in the study. PLWH with differing COVID-19 testing experiences/outcomes were selected to gather a wide range of perspectives, with the goal of having an equal number of participants in the following 3 groups: PLWH diagnosed with COVID-19, PLWH who tested negative for COVID-19, and PLWH who were not tested. PLWH who were tested for COVID-19 were tested using a nasopharyngeal sample for SARS-CoV-2 PCR. Only individuals with symptoms consistent with COVID-19 were tested. Results were usually available within 24–48 h.

To gather information related to COVID-19 experiences among PLWH, a semi-structured interview guide was created to understand the experience of PLWH during the COVID-19 pandemic. An additional file shows the qualitative patient interview guide that was developed for this study [see Additional file 1]. Questions were developed using the HIV and COVID-19 syndemic model [17] to explore psychosocial stressors and social determinants of health, while the Health Disparity framework [18] guided questions related to the healthcare system, including patient/provider factors. Interviews were conducted via telephone or Zoom by trained research staff in a private, secure location. Participants provided informed verbal consent and permission to audio record the interview. All interviews were recorded and professionally transcribed. This study was approved by the Institutional Review Board at the University of Chicago.

Transcripts were analyzed using Dedoose, a qualitative research software. The Framework Method for thematic analysis was used for creating and applying codes [19]. Each transcription was reviewed to ensure accuracy to the audio recordings, and a preliminary codebook was developed from the interview guide with clear definitions for each code to detect common themes. The codebook was then applied by the primary coder to five transcripts, and the secondary coders coded a subset of excerpts selected at random and achieved a kappa score of 0.80, establishing inter-rater reliability. Codes were then applied to all 32 transcripts and were reviewed by all coders (n = 3) for consensus of code application. Each transcript was coded iteratively and examined for emergent themes across transcripts. Themes were elicited based on grouping of code application, and representative quotes were selected to highlight significant themes.

## Results

Thirty-two patients participated in this study, with the majority being straight, Black, cis-gendered female-identifying people (Table 1). Of the 32 patients interviewed, 21 had been tested for COVID-19, 10 of whom tested positive for COVID-19. While each participant had unique experiences, some themes that were consistent among many patients are included in the following sections.

**Table 1:** Participant demographics (n = 32)

Characteristic	N (%)
Age (mean, SD)	39 ± 12
Gender	
Cisgender Female	17 (53.1)
Cisgender Male	15 (46.9)
Race/ethnicity	
Black/African American	29 (90.6)
Caucasian	2 (6.3)
Mixed Race	1 (3.1)
Comorbidities	
One or more present	20 (62.5)
Sexual Orientation	
Heterosexual	18 (56.3)
Gay	8 (25.0)
Bisexual	5 (15.6)
Pansexual	1 (3.1)
Test outcome	
Tested, COVID-19+	10 (31.2)
Tested, COVID-19−	11 (34.4)
Not Tested	11 (34.4)

SD, standard deviation

### Heightened fears of contracting COVID-19 due to perceived vulnerability

Many of the PLWH interviewed perceived their risk of contracting COVID-19 and likelihood of severity of COVID-19 infection to be influenced by their HIV status. Among those interviewed, 59% (19/32) perceived themselves to be more vulnerable to contracting COVID-19 compared to the general population. Of these participants, 89% (17/19) described their perceived vulnerability to COVID-19 to be due to immunosuppression related to HIV. Because of their HIV status, they assumed they were more likely to contract COVID-19.

“Really, really concerned, because with this condition I have [HIV], I’m more vulnerable. So, I was really scared. Yeah, I was really scared because I wasn’t sure. They said people with underlying conditions or people with low immune systems and things like that of that nature, so I was really afraid.”

[Cis-gendered African American female, not tested for COVID]

This participant described their reaction to receiving a diagnosis of COVID-19, with concern that they may develop more severe symptoms due to HIV.

**“**Heartbroken. I was a little nervous due to the fact that I already have another condition [HIV] that’s going on, and then to hear that this virus could affect those with a weaker immune system or anyone elderly. So, at first, it was a concern.”

[Cis-gendered African American male, COVID positive]

Of those who expressed concern that they were at increased risk for COVID-19, participants described perceived vulnerability as a result of not only living with HIV, but also due to essential worker status or not being able to isolate from others in their household who were not following social distancing recommendations. This participant, an essential worker, was worried about acquiring asymptomatic COVID-19 infection and potentially transmitting to others within their household:

“Very concerned because they considered us essential workers and we wasn’t allowed to just be at home. So, I was so afraid that I had to come out every now and then I had to go back and be home with my kids and possibly be a carrier with no symptoms and then they contract it with symptoms. I was afraid of that.”

[Cis-gendered African American female, COVID positive]

Another participant attributed their perceived higher COVID-19 risk to hosting an increased number of residents in their household due to loss of employment or housing during COVID-19. The increased number of persons in the multigenerational household created concern for increased exposure risk, particularly due to not knowing where their guests went during the day and not having enough space in their residence to accommodate everyone with social distancing:

“Because I don’t know where they’re going in the day and seeing through the day, and they’re coming in and sleeping in my house, just being in my house. And my mother’s here.”

[Cis-gendered African American male, COVID negative]

In contrast to those who believed themselves to be at higher risk for COVID-19, two participants felt that they would be protected due to their persistent use of antiretroviral therapy (ART).

“I almost thought I had it [COVID-19] at one point…And then, I heard that the retroviral drugs were being tested. And I thought, “Well, I have been on one for a very long time.” So I kinda convinced myself that being on that antiretroviral was somehow gonna help.”

[Cis-gendered Caucasian male, not tested for COVID]

Nine participants described anxiety and fear upon developing symptoms that could be consistent with COVID-19. Notably, two people experienced a general fear of receiving a positive test result and being unsure of what this meant for their health. Three of the participants indicated fearing for their life after receiving a positive test result and they were unsure of whether or not they would survive. These participants described a sense of helplessness and panic after learning of their COVID-19 diagnoses:

“I mean, in a time when you fear for your life, it’s bad. So, it was real scary and I just basically scared for my life and dying. Because I mean it’s out of my hands. Once they told me I had it, I couldn’t breathe. I couldn’t nothing but deal with the doctor, saying a prayer about it. Wasn’t nothing else for me to do.”

[Cis-gendered African American female, COVID positive]

Fear of COVID-19 infection was felt more acutely than that of other chronic illnesses. Further, COVID-19 exacerbated or brought on mental health challenges.

“I was very afraid of it [COVID-19]. Like I said before, I was more afraid of the COVID than I was the heart failure. That's what led to the panic attacks. Because they were saying—it really just keeps you in fear. There's just so much horrible you hear and death, death, death, death…And that's what I got in my psyche. It's death.”

[Cis-gendered African American male, COVID positive]

A participant also expressed fears that COVID-19 may decrease the effectiveness of ART and cause his HIV to progress.

“I was very concerned [about testing positive for COVID-19]. I was scared to death. My heart skipped because I thought that I could actually die. And with an underlying condition [HIV] and I immediately thought if I get it I’m gonna die. I was thinking what if COVID makes my Biktarvy not work? What if it makes it not work? And if HIV is—there’s another count with HIV and find out it was AIDS.”

[Cis-gendered African American male, COVID negative]

### Reactions to COVID-19 test results and medical mistrust

Of the 21 participants tested for COVID-19, most reported a positive experience with staff who made them feel safe and comfortable, and who were able to answer all of their questions. However, one person regarded their experience poorly because they felt as if the medical staff were not listening to what they were saying. This person expressed feelings of being manipulated and mistrust, stating “…I felt like they were trying to make me believe what they were saying.” [Cis-gendered African American female, COVID positive].

Of the 21 participants tested for COVID-19, participants who received a positive COVID-19 test result as well as participants who received a negative test result both described feelings ranging from relief to mistrust and skepticism upon learning their result. Five participants felt that they could not trust their test results. Two participants, one who received a positive test result and one who received a negative result, cited that they did not believe their diagnosis was accurate because it conflicted with what they believed to be happening with their health:

“I think I had it then, to be honest with you, I just didn’t think the test was accurate because, for my body to be hot like that, and I just felt like I was capturing everything they said how people would feel if they did have COVID.”

[Cis-gendered African American male, COVID negative]

This participant, who tested positive for COVID-19, expressed disbelief in her diagnosis of COVID-19:

“Yeah, because I kind of feel like they were trying to keep me there [the hospital] for—just for no reason. And I knew I didn’t have it. I knew I hadn't had no symptoms and I knew I did everything to stay out the way from getting any symptoms. And I feel like they were trying to almost put it on me like I had it, and I knew I didn’t. So, when I went home for 14 days and I came back for a checkup, they said everything was okay. So, I knew I didn’t have it at that point. I never was worried that I had it because my body tells me if I'm sick like that.”

[Cis-gendered African American female, COVID positive]

Among other participants who received a positive diagnosis but who did not initially believe their test results, reasons for disbelief ranged from anxiety and awareness of the severity of COVID-19 to disbelief in the virus itself, and were independent from their HIV status: “I didn't think that the COVID-19 was real, that’s why I was feeling so shocked that I had it.” [Cis-gendered African American male, COVID positive].

### Impact of COVID-19 and HIV diagnoses

Eight out of the ten PLWH who tested positive for COVID-19 regarded their HIV diagnosis as having a more profound impact on their lives as compared to the COVID-19 diagnosis. Seven of these 8 participants attributed HIV as the more profound diagnosis because of the permanence of HIV, whereas one can recover from COVID-19. While this same participant described fear and panic of the acute impact of COVID-19 on their health, they felt that HIV had a greater long-term impact on their life and health: “The COVID is a pandemic. And you can get rid of COVID. You can’t get rid of HIV. So, that’s how I would differ. I’m still dealing with HIV and heart failure. I’m not dealing with COVID anymore.” [Cis-gendered African American male, COVID positive].

Another participant highlighted how her overall knowledge of COVID-19 and its potential short-term outcomes made it an easier diagnosis to process than HIV:

“So, finding out I was COVID, COVID is something that can either take your life or you can move on from it. So, I was confident that I was gonna get better. Finding out that I was HIV-positive—because I was young and I was terrified, I wasn't educated about HIV, so I was scared. And to me, it just said death. So, once I was diagnosed with it, it was then I learned about it. And learning about it made me more comfortable with it because it wasn't, "Oh, I'm gonna die tomorrow." That was my perception before I knew about it.”

[Cis-gendered African American female, COVID positive]

Two of the ten PLWH who tested positive for COVID-19 regarded their COVID-19 diagnosis as having a more profound and immediate impact on their lives. Both participants attributed this to a fear of immediate morbidity and mortality as a result of unpredictable COVID-19 outcomes. While HIV still had a profound impact on their lives, these participants felt that they had support and could manage living with HIV because it had been researched and understood for many years. This participant commented on the fact that treatment of HIV is both well understood and manageable, which led him to feel secure in his HIV prognosis. However, this patient received his positive COVID-19 diagnosis at a time when there were many unknowns about the virus itself and treatment options.

“Yeah, well, with the HIV, that’s a done deal. With COVID-19, it’s not a done deal for everyone that gets it. I don’t know why some die and some don’t die, but it’s not really a done deal like HIV is. HIV is a done deal.”

[Cis-gendered African American male, COVID positive]

Similarly, this participant felt that the uncertainties and high death toll of COVID-19 made this a more concerning diagnosis than HIV, particularly with this patient’s symptoms.

“But for some reason the COVID took more because one, the statistics of people dying and stuff, that really hit home because I considered that worse than my HIV for apparent reason. I did because nobody seems to know what it is, how can it affect you. They don’t have any clear picture of it. That was more scary than my HIV because it does work on your respiratory system, and I was having a hard time breathing even every time I had to go to the restroom even though it was a short distance. I had to come off the oxygen and before I could stand up, I had to try to catch my breath first, and then walk to the bathroom. So, that was definitely more scary than me receiving my HIV status.”

[Cis-gendered African American female, COVID positive]

### Stigma associated with HIV and COVID-19

PLWH commented on the HIV-related stigma they have experienced, with several participants comparing the potential stigmatizing experiences associated with a person receiving a COVID-19 diagnosis with those related to living with HIV. This participant expressed fears of experiencing compounded discrimination as a result of both HIV and COVID-19.

“I’m concerned about, first of all, about the inequality in treatment. Because you know, I’ve already faced that just with the diagnosis that I have [HIV]. I’ve already faced that in many settings. And then, with that hanging in the balance, that’s something else [COVID-19] to be discriminated against for.

[Cis-gendered African American female, not tested for COVID]

Similarly, another participant described her belief that having HIV would result in subpar care for those who were diagnosed with COVID-19:

“Absolutely, because I felt like once you were identified, they would be like, ‘Okay. Well, they on their way out the door anyways, so let them go ahead...’ I don’t think we would receive the best care because people think, ‘oh, what’s the use?’”

[Cis-gendered African American female, not tested for COVID]

This participant drew further parallels between HIV and COVID-19 related discrimination:

“The same way that people see, the same stigma that we were getting, people with [HIV], the same treatment that we were getting, people that had COVID or people that were survivors of COVID, they are getting that same stigma…People don’t want to be around them. People don’t want to eat off the same things as them. People don’t want to smoke a half a cigarette after them or anything like that because there’s that same stigma that, “Is this gonna give it to me? If I eat off the same spoon as this person, is it gonna give it to me?” And so, I think a lot of cases of that group of people are now going to be able to be more informed. They’re gonna be able to be more aware and they’re gonna be able to be in the shoes of a person who may have HIV or of a person who may have cancer. That same stigma that we were getting, that same hatred and negativity, a lot of people who are survivors or who are living with COVID-19 are having that same stigma, which is one of the things that I noticed.”

[Cis-gendered African American male, COVID negative]

In addition to drawing parallels between HIV and COVID-19 related stigma, this participant expressed hope that if a larger proportion of the population experiences stigma, it can help to alleviate HIV-related stigma by allowing persons to “be in the shoes of a person who may have HIV,” and perhaps develop a more tolerant and understanding societal approach toward PLWH.

## Discussion

In this study, the majority of PLWH reported having an increased perceived vulnerability to COVID-19 transmission due to living with HIV as a pre-existing condition and experienced concerns of increased morbidity or mortality should they contract COVID-19. The majority of people interviewed in this study identified as Black, reflecting a population on the southside of Chicago that is particularly vulnerable to COVID-19 [20, 21]. However, some individuals noted a perceived protection from COVID-19 due to their persistent use of ART as a result of early reports of possible protective effects of certain ART regimens [22]. The heightened notion of perceived vulnerability among PLWH is understandable due to the constant, and sometimes unclear, emphasis of underlying medical conditions in regard to COVID-19 [23]. However, several studies have demonstrated that PLWH are not at an increased risk of contracting COVID-19 when compared to the general population [24, 25]. Nevertheless, despite this growing evidence, PLWH remain concerned about their vulnerability to COVID-19, which can play a role in anxiety and other mental health disorders and contribute to added stress and isolation of the COVID-19 pandemic for PLWH [17, 26].

Overall, PLWH had many fears regarding COVID-19 testing that mostly stemmed from the apprehension of receiving a positive diagnosis and not knowing how that diagnosis might impact their health and wellbeing. A recent qualitative study examining the psychological experience of hospitalized COVID-19 patients found that patients’ attitudes toward COVID-19 included fear, denial, and stigma in the early stages, which gradually developed into acceptance in the later stages [27]. Accompanied by the medical fears are potential stigmas for testing positive for COVID-19, from an “enacted” stigma in which patients experience explicit discrimination, to “felt-normative” stigmas in which patients may feel internalized stigma as if they did something wrong by contracting an infectious disease [28]. Participants also expressed fear of experiencing aggravated stigma from COVID-19 in addition to HIV-related stigma. Internalized stigma contributes to decreased healthcare engagement, which may be exacerbated when an individual is faced with multiple stigmatizing health conditions, such as HIV and COVID-19, in addition to often facing intersectional stigma related to racial, sexual, and gender minority identities [6, 29]. It is crucial for public health messaging to avoid further stigmatizing conditions and the behaviors associated with transmission, as we have seen with HIV, which can exacerbate inequities and hamper the public health response [26].

The majority of PLWH interviewed in this study had positive interactions within the healthcare system and during COVID-19 testing, yet a significant proportion of participants expressed concerns that they could not trust the test results. Medical mistrust has been described as a social determinant of health and is considered to be a result of historic and ongoing structural injustices that disproportionately impact Black patients, with downstream health impacts [30, 31], including for HIV [32, 33]. Mistrust has been seen throughout the United States during the COVID-19 pandemic due to misinformation and politicization of the COVID-19 response [34]. Furthermore, it is possible that some of the PLWH in our sample had developed mistrust of the healthcare system over time during their numerous HIV clinical encounters that was exacerbated during the COVID-19 pandemic. For PLWH, misinformation and lack of trust could lead to avoiding COVID-19 testing altogether or not fully understanding the implications of a COVID-19 diagnosis. Furthermore, this mistrust could also result in COVID-19 vaccine hesitancy or increased distrust of the medical and public health systems during future health crises.

There are some important limitations in this study to consider. All participants were recruited from The University of Chicago, a large academic medical center that remained operational during the COVID-19 pandemic. Thus, our findings may not reflect the experience of patients who receive HIV care at smaller or rural healthcare clinics. In addition, the interviews were conducted during the relatively early stages of a rapidly evolving pandemic, representing the experiences and feelings of PLWH at the time of data collection without accounting for how their perspectives may have changed over time. The comparison of COVID-19 and HIV diagnoses may have been affected by the COVID-19 testing status of participants, as those who tested positive and recovered from COVID-19 likely experienced a decreased sense of fear surrounding the uncertainty or high mortality rate related to COVID-19 when compared to those who were not tested or who tested negative. Similarly, it is possible that those without HIV also felt a sense of stigma when tested for or diagnosed with COVID-19, yet PLWH may have been better able to recognize potentially stigmatizing experiences and demonstrate the fear of aggravated stigma. Lastly, various sources of inevitable bias associated with qualitative research may have impacted study findings. PLWH who have better engagement in the healthcare system may have been more likely to participate than others, and interviewer presence may have impacted participant responses or the selection of relevant themes during data analysis. However, establishing inter-rater reliability diminished the effects of bias during qualitative data analysis and led to consensus on application of codes and elicitation of significant themes.

## Conclusions

During a time of heightened fear and concern related to the COVID-19 pandemic, PLWH have the added burden of considerable perceived vulnerability due to HIV. This fear affects not only their physical and mental health, but also their sense of societal stigmatization. Because the results presented in this study were gathered relatively early in the pandemic, additional research should be conducted in the future to identify potential long-term effects of the COVID-19 pandemic on PLWH, as well as attitudes and experiences among PLWH related to COVID-19 vaccination. For the next phase of the pandemic, it is crucial to address any medical mistrust, especially among vulnerable populations and PLWH who are less engaged in the healthcare system. The findings presented here can help both physicians and public health entities better understand the experiences and concerns of PLWH and work to find ways to decrease fear, generate trust, and improve holistic care during the pandemic and beyond.

## Supplementary Information

**Additional file 1:** Patient Interview. Semi-structured qualitative patient interview guide developed for study.

## Data Availability

Data cannot be shared due to confidentiality concerns, as patients may be identifiable.

## Abbreviations

COVID-19: Coronavirus disease 2019
HIV: Human immunodeficiency virus
PLWH: People living with HIV
ART: Antiretroviral therapy
