# Supplementary material for: “That same stigma...that same hatred and negativity:” a qualitative study to understand stigma and medical mistrust experienced by people living with HIV diagnosed with COVID-19
Source: BMC Infect Dis. 2021 Oct 14;21:1066. doi: 10.1186/s12879-021-06693-5 (PMC8515148; doi:10.1186/s12879-021-06693-5)
Supplement: Supplementary file 1 — Additional file 1: Patient Interview. Semi-structured qualitative patient interview guide developed for study. [file 12879_2021_6693_MOESM1_ESM.docx]

*Patient Interview*

**PROTOCOL TITLE: Living with HIV in the Era of COVID-19**

Date: __________________________________

Patient ID: __________________________________

Interviewer ID: __________________________________

COVID-19 Status: Not Tested Tested Negative Tested Positive

**Interview Objectives**

· Determine the experiences of PLWH during the COVID-19 pandemic

· Identify strategies to better support PLWH in staying safe and accessing appropriate medical care

· Identify forms of medical communication that work during times of shelter-in-place

**PART 1. Introduction**

**· Quick review**

**o Thank you for taking the time to take part in this interview. We hope that it will help us better understand the experiences of those living with HIV during the COVID-19 pandemic and help us find ways to better support people living with HIV.**

**o We are going to be asking a lot of questions about your health and your experiences during the pandemic. If there are any questions you would rather not answer, please let me know and we can skip that question and move on.**

**o We would like to do audio recording, which is for research purposes and for internal use only. When we are done with this interview, someone will type out every word that was said. After that, we will destroy the recordings so that everyone’s participation is anonymous and confidential.**

**o We will be asking lots of questions. There are no right or wrong answers; we are just interested in your opinions and your experiences. After responding, we may ask you “why” – not because we disagree, but to make sure we understand your thoughts.**

**Do you have any questions before we begin?**

**I’ll be taking notes during the interview. However, with your permission, I’d also like to audio-record our conversation as I may not be able to write down everything quickly enough. The audio recordings will be destroyed at the end of the study and your name and voice will not be connected to this information.**

**Do I have your permission to record our conversation? YES NO**

**Consenting Participant: I’ll be reading you a verbal consent, after which I will start the recording**

**PART 2. Demographic Information**

**We are going to ask you a few questions about yourself.**

1. What is your age? ____
2. What is your gender?

***Probe:*** *Does your current gender match the gender you were when you were born? Options: cisgender female, cisgender male, transgender female, transgender male, non-binary, queer, other*

1. What is your sexual orientation?

***Probe:*** *options include Lesbian, gay, straight, queer, questioning, asexual, pansexual*

1. What is your race?
2. Do you identify as Hispanic/Latinx?

**PART 3. General Information**

1. On March 12, the World Health Organization declared COVID-19 a pandemic. We know that everyone’s lives are different now. Can you tell me about how the COVID-19 pandemic has impacted your life in general?

***Probes:*** *employment impact – current employment status; financial strains; childcare/home-schooling responsibilities; mental health; physical health; relationship strains; food access*

1. Due to statewide stay-at-home orders and social distancing guidelines, have you experienced any significant changes to your living situation? What is your current living situation?

***Probes:*** *number of people in home; experience living at home during this time*

1. How would you say these orders have impacted your social life?
   1. How has mental health been affected?
   2. If you drink alcohol or use drugs, how has that changed during the pandemic?

***Probes:*** *unable to see family/friends; psychological issues – anxiety, depression; increased feelings of social isolation and/or loneliness; anxiety about health*

1. What steps are you taking to stay safe during this time (that were not already mentioned before)?

***Probes:*** *wearing a mask; family members bringing groceries; using a meal service*

1. Can you please describe what your HIV care routine was like before the pandemic?

***Probes:*** *relationship with provider; how often do you see your provider; how easy/difficult are these visits; medication access*

1. How has the COVID-19 pandemic impacted both your ability to remain in HIV care?
   1. What about the overall quality of your medical care?
   2. Have you encountered difficulties staying on your medication? If yes, why?

***Probes:*** *change in medical care - worse, better, or just different and why; difficulty accessing medication; adherence to ART; change in health coverage/insurance; fear of attending medical appointments; mental health – anxiety, depression; financial strain; telemedicine*

1. Is there anything you can think of that would make your HIV-care experience better/easier during the pandemic?
2. How has the COVID-19 pandemic impacted your sex life and/or romantic relationship(s)?

***Probes:*** *relationship status; having sex less often or more often; being away from partners; not having access to birth control/condoms; unable to meet anonymous partners/increased meeting of anonymous partners*

1. At the beginning of the pandemic, how concerned were you about contracting COVID-19?

***Probes:*** *not worried, very concerned, or unsure about likelihood of contracting COVID-19; general fear vs. fear due to having HIV; any significant changes due to these fears* – *adjusting living situation, not attending medical appointments, limiting contact with others, avoiding crowds, etc.*

1. As the pandemic worsened, did your level of concern change?
2. Are you currently dealing with any other health issues in addition to HIV?

***Probes:*** *comorbidities - diabetes, hypertension, etc.; medications*

**PART 4. Symptoms and Testing**

1. Was there ever a time in the past few months where you thought you might have COVID-19?

***Probes****:* *why? what symptoms? exposures to other people with COVID-19?*

1. **If yes**, can you talk me through what happened when you first started experiencing symptoms related to COVID-19?

***Probes:*** *what kind of signs and/or symptoms; mental health – anxiety, fear; were you immediately concerned about having COVID-19; how did you recognize these were COVID-19 symptoms – Internet, news, COVID-19 triage line/PCP or another medical provider?*

1. What were some of the fears you had when you were experiencing these symptoms?

***Probes:*** *health concerns due to HIV; living situation – possibly infecting family members/roommates/friends; finding somewhere to be tested; accessing health care safely without infecting others*

1. After you began experiencing these symptoms, were you tested for COVID-19?

*Probes:* **if yes** – *how long did you wait to look into getting tested for COVID-19?*

--- --- ---

*Probes:* **if no** – *why not?*

1. **If no**, are you still concerned that you had COVID-19 at some point, or that you might get it in the future?

***Probes:*** *how does that concern/fear impact your life, especially as someone living with HIV? how do you think it will continue to impact your life throughout the pandemic?*

**PART 5. COVID-19 Testing Experience – Only ask if answered “yes” to question about being tested**

1. How did you determine that you needed to be tested for COVID-19?

***Probes:*** *called COVID-19 triage line/healthcare setting – clinic or hospital/PCP or another medical provider; went to the ER – was it at a medical facility where you had received care previously; individual choice – had certain symptoms and went to get tested without consulting anyone*

1. What concerns did you have about going to get tested for COVID-19?

***Probes:*** *financial concerns, infecting others, finding a testing center, etc.*

1. Did you have any fears/stigma regarding being tested for COVID-19?

***Probes:*** *concerns about unjust treatment when accessing/attempting to access the healthcare system, privacy/confidentiality, etc.*

1. Can you describe your overall testing experience? What made it a good or bad experience?

***Probes:*** *where did you get tested; how long did you wait to get tested; how long did the test take; how invasive was the test; did you feel safe when getting tested; did you feel like you were treated fairly when getting tested; did the medical provider answer any questions you had, take the time to make you feel comfortable, etc.*

1. Can you talk about how you felt when you received your test results?

***Probes:*** *how long did it take to get the results; did you trust the results?*

*--- --- ---*

*Probes:* **if positive** – *what was your reaction; did you feel confident in knowing the steps*

*you needed to take to recover at home vs. when you would need to see a doctor or go to the ER?*

--- --- ---

*Probes:* **if negative** – *what was your reaction; did you feel relieved; did you feel the need to be re-tested?*

1. **If negative**, are you still concerned that you had COVID-19 at some point, or that you might get it in the future?

***Probes:*** *how does that concern/fear impact your life, especially as someone living with HIV? how do you think it will continue to impact your life throughout the pandemic?*

**If positive:**

1. How did this result compare to receiving your HIV diagnosis?

***Probes:*** *similarities and differences; compare reaction/feelings – any trauma, PTSD, etc.*

1. Were you hospitalized? If yes, please tell me about that experience.
   1. Is there anything that could have been done to make that experience better?

***Probes:*** *Length of stay, do you feel you were given appropriate care; were you housed with other patients - how many; did you feel like you were treated as fairly/respectfully as possible?*

1. When you returned home, how did it feel to be living with your COVID-19 diagnosis? How did social distancing and your lifestyle further change while you were recovering in self-isolation?

***Probes:*** *how many days/weeks were you in self-isolation; were you able to receive support during this time; did your medical provider perform follow-up visits over the phone; did you ever need to seek further medical attention at your doctor’s office or ER?*

**PART 6. Implementation Changes Related to HIV and COVID-19**

1. How do you think having HIV has impacted your overall experience with the healthcare system during the pandemic?

***Probes:*** *unable to access health care; concerns about privacy/confidentiality; concerns about unequal treatment in the healthcare system; adjustments to standard HIV visit schedule/format (not sure when would see doctor)*

1. Are there any changes you think medical systems can make to help HIV patients access healthcare safely?

***Probes:*** *telemedicine, mobile pharmacy, mail-order/delivery pharmacy, etc.*

1. How do you think physicians can improve practicing medicine and communicating with their patients during this time?

***Probes:*** *telemedicine – any concerns about meeting with a physician over the phone/video rather than in-person; social distancing in-person when possible*
